# Vaccine acceptance, determinants, and attitudes toward vaccine among people experiencing homelessness: a systematic review and meta-analysis

**DOI:** 10.1186/s12879-023-08878-6

**Published:** 2023-12-15

**Authors:** Dung Anh Nguyen, Habib Olatunji Alagbo, Toka Adel Hassan, Leonardo D. Mera-Lojano, Esraa Osama Abdelaziz, Nguyen Pham Nguyen The, Abdelrahman M. Makram, Omar M. Makram, Randa Elsheikh, Nguyen Tien Huy

**Affiliations:** 1 Health Science Department, University of The People, Pasadena, CA USA; 2 Online Research Club, Nagasaki, Japan; 3 V.N, Karazin National University, Kharkiv, Ukraine; 4 Faculty of Medicine, October 6 University, Giza, Egypt; 5 ASOCEM UCE - Scientific Association of Students of Medicine, School of Medicine, Faculty of Medical Science, Central University of Ecuador, Quito, Ecuador; 6 Faculty of Pharmacy and Biotechnology, German University in Cairo, Cairo, Egypt; 7 University of Medicine and Pharmacy at Ho Chi Minh City, Ho Chi Minh City, Viet Nam; 8 School of Public Health, Imperial College London, London, UK; 9 Center for Health & Nature, Houston Methodist Hospital, Houston, Texas 77030 USA; 10 Deanery of Biomedical Sciences at Edinburgh Medical School, University of Edinburgh, Edinburgh, UK; 11 School of Tropical Medicine and Global Health, Nagasaki University, Nagasaki, Japan

**Keywords:** COVID-19, Vaccine hesitancy, Homeless, Attitudes

## Abstract

**Background:**

COVID-19 has caused millions of deaths globally, with vulnerable populations such as people experiencing homelessness (PEH) at higher risk. This systematic review and meta-analysis aims to identify the prevalence and key factors contributing to vaccine acceptance experienced by PEH.

**Methods:**

The protocol of this study was registered in PROSPERO (CRD42023391659). We included studies that reported relevant information about vaccine acceptance or vaccine hesitant/refusal among PEH. Eight databases were systematically searched in January 2023. Meta-analysis was conducted for the prevalence of vaccine acceptance, vaccine uptake, and factors associated with vaccine acceptance. Attitudes toward vaccines were combined into bar charts.

**Result:**

A total of 29 papers were included in this systematic review and 19 papers were included for meta-analysis. The pooled prevalence of COVID-19 vaccine acceptance among PEH was 66% (95%CI: 58%-73%). Our meta-regression showed vaccine acceptance was significantly increased over time. Moreover, subgroup meta-analysis showed that PEH were more likely to accept the COVID-19 vaccine after June 2021 (78%, 95%CI: 65%-86%) compared with earlier period (56%, 95%CI: 54%-59%). Subgroup meta-analysis also revealed that women and participants without underlying medical condition (chronic diseases) were significantly less likely to accept the COVID-19 vaccine, compared to men and those with medical conditions, respectively.

**Conclusion:**

The study emphasizes the need for targeted public health interventions aimed at increasing vaccine acceptance among PEH, especially at the early stage of the pandemic, among females, those without underlying medical conditions, being Black (in Canada and the USA), and young people. These interventions should address the common concerns of vaccine safety, adverse effects, effectiveness, and distrust in health care systems. In addition to offering vaccinations in different areas convenient to them, education programs could be established to increase vaccine acceptance among PEH.

**Supplementary Information:**

The online version contains supplementary material available at 10.1186/s12879-023-08878-6.

## Introduction

The COVID-19 virus has positioned itself as a pandemic that has taken away millions of lives worldwide. As of May 2023, there are nearly 7 million deaths [1, 2]. Despite health measures taken to reduce the risk of transmission, the virus is still a health threat, especially for vulnerable groups such as people experiencing homelessness (PEH) [3]. Although massive amounts of COVID-19 vaccination doses are spread worldwide, not all people are yet vaccinated. Moreover, some people are still reluctant to receive the vaccine [4, 5]. This behavior is influenced by many factors, such as trust in the government, complacency and convenience toward vaccine producers, adverse effects, etc. [6].

High acceptance rates are essential for achieving herd immunity and ending the COVID-19 crisis, while hesitancy can lead to increased transmission and prolonged health and economic impacts. Vaccine hesitancy poses a great risk to PEH, as they appear to be at a higher risk of infection, morbidity, and mortality compared to the general population [7, 8]. In general, homelessness and unstable housing have been found to be associated with poor health conditions [9]. PEH are especially susceptible due to limited access to healthcare and the inability to socially distance [3]. In addition, they are often older and have underlying chronic illnesses.

It is plausible that PEH may exhibit lower COVID-19 vaccination rates compared to the general population, sharing common factors of vaccine hesitancy. These factors include concerns about severe side effects, mistrust in vaccine ingredients, skepticism toward government authorities, gender-related differences (with women showing higher hesitancy), cohabitation with a partner, and lower levels of health education [8, 10, 11]. Nevertheless, further research is required to identify the leading risk factors for vaccine hesitancy in PEH. Understanding the reasons underlying vaccine hesitancy is essential to develop appropriate strategies to increase vaccination acceptance and thereby improve health prognoses for these groups.

Hence, the objective of this systematic review and meta-analysis is to identify the prevalence and factors associated with vaccine acceptance among PEH and the vaccination constraints experienced by them. In addition to the COVID-19 vaccine, other kinds of vaccines will also be included in this review to provide a more comprehensive understanding of the challenges encountered by PEH in accessing and accepting vaccines.

## Methods

### Protocol registration

Our protocol was registered on 13 January 2023 by PROSPERO under registration number CRD42023391659 with the purpose of “(estimating) the prevalence and associated factors of vaccine hesitancy and vaccine refusal among homeless people” [12]. We followed the PRISMA guidelines [13] and Tawfik et al.’s paper [14] of the 14 steps to conduct a systematic review and meta-analysis. The PRISMA checklist is reported in Supplementary file 1.

### Selection criteria

The inclusion criteria were as follows: (1) any primary study reporting prevalence or associated factors of vaccine hesitancy or vaccine refusals among PEH, including residents of homeless shelters or non-shelters; (2) no restrictions on language, ethnicity, gender, geography, or socioeconomic status; and (3) no restrictions on study design. The exclusion criteria were as follows: (1) articles without full text available; (2) conference papers, letters, commentaries, news pieces, editorials, author responses, and books; and (3) studies with data not reliably extracted which means different data between abstract and results, duplicated, or overlapping datasets.

### Search strategy

On 14th January 2023, we searched articles on PubMed, Scopus, EMBASE, Web of Science, Google Scholar, Cochrane, ClinicalTrials.gov, and metaRegister of Controlled Trials (mRCT) with the main search strategy: (homeless OR homelessness OR “ill-housed” OR unhoused OR shelter OR shelters OR unsheltered OR "street people" OR “street person*” OR “insecure housing” OR vagabonds OR hoboes) AND (willingness OR readiness OR hesitancy OR hesitancies OR delay OR delays OR hesitant OR refusal OR refusals OR acceptance OR accept OR reluctance OR agreement OR undecided OR indecisive OR indecisiveness OR indecision OR uncertain OR uncertains OR skeptic OR skeptics OR doubt OR doubts OR decline OR declines) AND (vaccine OR vaccines OR vaccination OR immunization OR immunisation OR immunized OR shot OR shots OR booster). For details of the specific syntax used in each database, please refer to Supplementary file 2.

### Title/abstract screening

The screening process was carried out by two independent teams, with three members in each team. Independent reviewers must decide between “include”, “exclude”, or “maybe” for each title/abstract. After that, each group discussed the final decision. In case of conflicts or disagreements, papers were automatically included for the full-text step.

### Full-text screening

In this step, we read more carefully to find the articles that met our criteria and excluded unqualified articles; in particular, in this step, we did not have the “maybe” option for our results. Our team worked the same as the previous step with two teams having three members working independently and having a discussion for the final decision. However, this time, any conflicting results after the discussion were resolved by our supervisor (AMM).

### Manual search

The manual search was conducted on 15th February 2023 to check for missing articles by checking references, citations, and similar articles of the included studies on PubMed, Scopus, EMBASE, and Web of Science. The manual search results were subjected to the same full-text screening process. After the analysis, we conducted searches again on a few databases, in cases any papers were published after the initial search.

### Data extraction process

Data extraction was conducted according to a predefined data extraction table, which was developed using the five most compatible studies found by the systematic search. We summarized the following data from the included studies: authors, year of publication, country, study design, kind of vaccine, type of homeless, sample size, number of people already vaccinated, time of survey, population characteristics, study objective, outcome measures, study limitation, and main finding. Then, we extracted the following data: sociodemographic (sex, race/ethnicity, age, education level, health insurance, duration of homelessness, source of COVID-19 information) and main outcomes (kind of vaccine, acceptance rate, hesitancy rate, refusal rate, attitudes toward vaccination, barriers to accessibility, and factors associated with vaccine acceptance variables). This step was performed in the same fashion as the screening processes. There were at least two reviewers independently extracting the same paper. Extracted data were cross-checked by leaders, and discrepancies were solved by a discussion with all involved reviewers.

### Quality and risk of bias assessment

After data extraction, the quality of the included studies was assessed using five modified questions, which were influenced by the "Quality Assessment Tool for Observational Cohort and Cross-Sectional Studies" of the National Heart, Lung, and Blood Institute [15]. Five questions were (1) Was the research question or objective in this paper clearly stated? (2) Was the study population clearly specified and defined? (3) Were the outcome measures (dependent variables) clearly defined, valid, reliable, and implemented consistently across all study participants? (4) Is it clear what was used to determine statistical significance and/or precision estimates? (5) Does the response rate raise concerns about non-response bias? Non-response should be < 40%).

Each question was marked by 1 for a “yes” answer and 0 for a “no” answer. Then, we summed and defined the quality with more than 4 points as good, 3 points as fair, and less than 3 points as poor. Each reviewer performed this step independently, and conflicting results were resolved by discussion.

### Data analysis

Data were grouped according to the kind of vaccine. If more than three studies reported the event rate (prevalence) of vaccine acceptance and vaccine uptake in homeless people or risk factors for vaccine hesitancy, proportional meta-analysis was estimated by standard meta-analysis using the meta package in R project software. Participants who have already received the vaccine and those who are willing to receive the vaccine will be coded as acceptance. While only those who already received the vaccine will be coded as vaccine uptake. The prevalence of vaccine acceptance and uptake were computed with the corresponding 95% confidence intervals (95%CI). Heterogeneity was considered statistically significant if I^2^ values > 50%.

A meta-regression was performed to analyze the relationship between the time interval and COVID-19 vaccine acceptance. The time interval was measured in days, with the final date of the survey duration serving as the time reference. If there was a significant association (*P* < 0.05) between the time interval and vaccine acceptance, a specific time point subgroup meta-analysis for COVID-19 vaccine acceptance was conducted to reduce heterogeneity. A subgroup meta-analysis for a time point was considered feasible if the I^2^ value for the subgroup was below 40%.

If more than 3 papers report raw data for the same associated factor variables, then a meta-analysis will be conducted for odds ratios. Meta-analysis will be estimated by Review Manager 5.4.1 software. Odds ratios will be computed with the corresponding 95%CI—heterogeneity was considered statistically significant if I^2^ values were > 50% and the *p*-value significance cut-off was 0.05.

There is one paper [16] for which we could not obtain the raw data for homeless shelters and homeless non-shelters, so we counted a portion of migrants as PEH. The migrant population in this paper exhibited similar characteristics to the homeless population, leading us to combine them for the purposes of analysis. We also assumed that some missing values for vaccine acceptance/hesitant events were random, so any missing value was removed from the analysis.

## Results

### Study characteristics

**Fig. 1:**
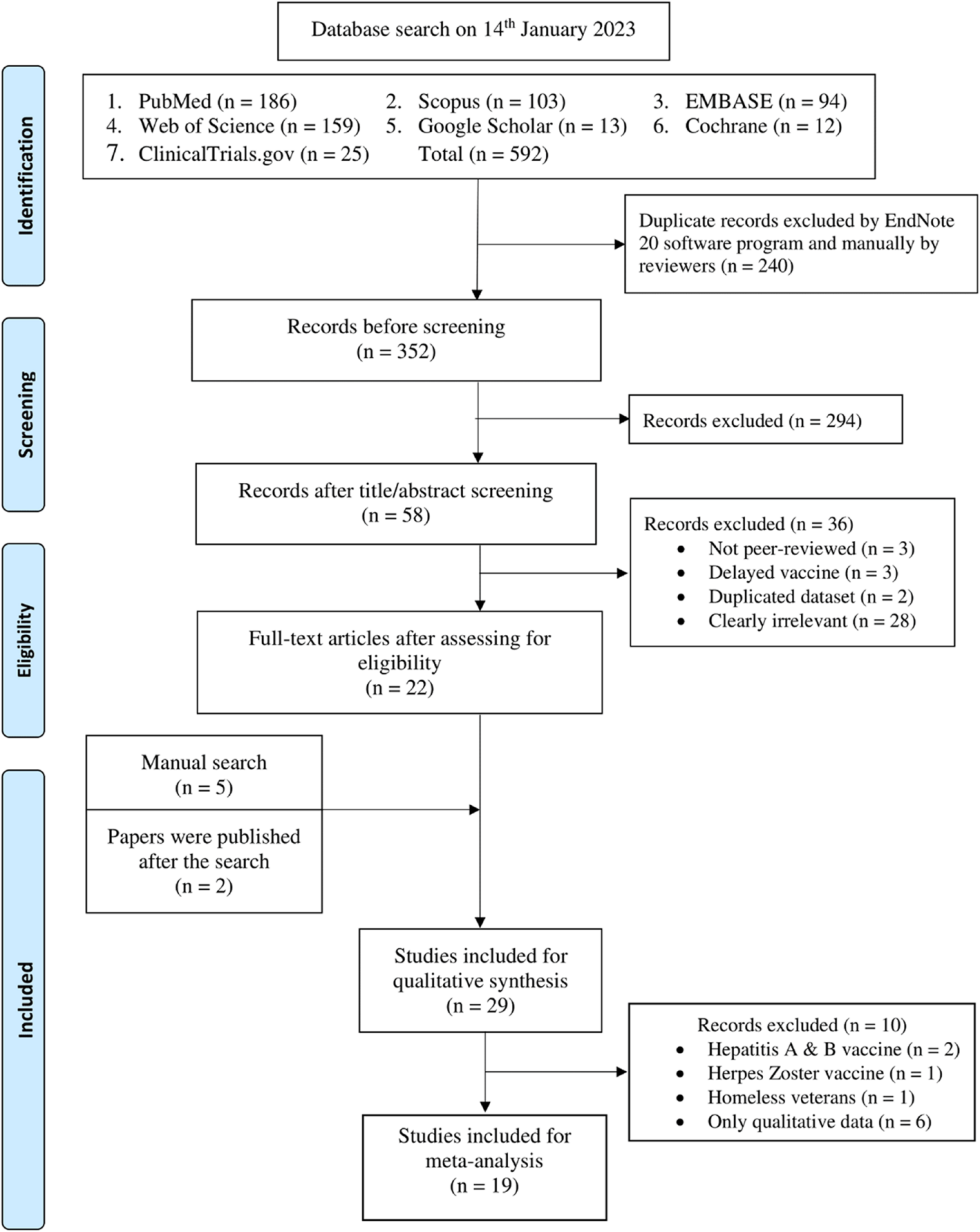
PRISMA flowchart for the inclusion of studies

Our initial search yielded 592 articles, from which 240 duplicates were removed. Another 294 were excluded after title and abstract screening, and an additional 36 were excluded after full-text screening. Seven articles were identified manually, resulting in a total of 29 articles included in the systematic review and 19 for the meta-analysis. For the PRISMA flowchart for the inclusion of studies, please refer to Fig. 1.

Our systematic review identified 19 studies from the USA [11, 17–34], three studies from France [10, 16, 35], two studies from Australia [36, 37], two studies from Italy [38, 39], one study from Canada [40], one study from England [41], and one study from Germany [42]. Two prospective cohort studies examined Hepatitis A and B vaccination [29, 36]. All other studies are cross-sectional; of which one investigated the Hepatitis A vaccination [18], three investigated Influenza vaccination [28, 35, 41], one investigated Herpes Zoster vaccination [24], one investigated the 7-vaccine series for baby [21], and all other studies examined the COVID-19 vaccination.

For quality assessment results, 12 studies were considered good, 12 studies were considered fair, and five studies were considered poor – for more details of quality assessments, please refer to Supplementary file 3. The characteristics of the COVID-19 studies are presented in Table 1, and other vaccines (not COVID-19) are presented in Table 2.

**Table 1:** Characteristics of the study about COVID-19 vaccine acceptance sorting by time

Author/year/country	Date of survey	N	Target population	Outcome measures	Acceptance rates	Predictors of acceptance	Predictors of hesitance	Quality
Longchamps/2021/ France [10]	May 2—June 28 2020	235	Homeless shelters	Acceptance: “If a vaccine existed would you be willing to get vaccinated?”	59.15%		1, Being women 2, Living with partners 3, Having legal residence 4, Having low health literacy	Good
Hsu/2021/USA [23]	August 7—September 17 2020	78	Young PEH	Acceptance: “When COVID-19 vaccine is available, how interested would you be in taking the vaccine?” in 7-level scale Attitudes: Respondents were asked their level of agreement on whether they believed the potential COVID-19 vaccine would (1) be necessary to protect their health, (2) do a good job to stop the COVID-19 spread, and (3) be safe	56.41%			Fair
Swendeman/2022/USA [33]	October 2020	153	Young PEH	Acceptance: “What is the likelihood that you will get a COVID-19 vaccination when it is available?”, on a 5-level scale (‘Very likely’, ‘Likely’, ‘Somewhat likely’, ‘Not likely’, or ‘Refuse to answer/don’t know’) Attitudes: VHS	51.63%			Fair
Knight/2021/USA [11]	July—October 2020	91	PEH	Interview by telephone		1, Return to regular life 2, Wait until other take the vaccine	1, Need more data of vaccine 2, Negative experiences with other vaccines 3, Mistrust in government	Poor
Iacoella/2021/Italy [39]	February 1—February 15 2021	112	PEH	Acceptance: Would you be willing to be vaccinated against COVID-19?	64.29%		1, Being female	Poor
Meehan/2022/USA [27]	February 9 – February 23 2021	106	Homeless shelters	Attitudes: open-ended questions	57.55%	1, Protect own health 2, Protect others 3, Resume travel and social activities	1, Side effects concerns 2, Vaccine is being new 3, Human experiments 4, Don’t trust medical field	Fair
Kuhn/2021/USA [25]	December—February 26 2021	90	PEH	Acceptance: respondents were asked if they would take the vaccine if they were offered it, with possible responses of “yes,” “no” or “prefer not to answer Attitudes: could not determine	52.22%	1, Trusting COVID-19 information from the official sources	1, Having lower COVID-19 threat index scores 2, Highly engage in protective behaviors 3, Trusting personal contacts for COVID-19 information	Fair
Rogers/2022/USA [31]	November 1—February 28 2021	672	Homeless shelters	Acceptance: “Once a vaccine against COVID-19 becomes available to you, do you plan to get it?”	53.72%	1, Higher level of educational attainment 2, Receiving Influenza vaccine before	1, BeingMultiracial 2, Being Black/African American 3, Being female 4, Safety concerns 5, Need more information 6, Not afraid of COVID-19	Good
Rodriguez/2021/USA [30]	December 20—March 7 2021	84	Homeless patients visited emergency departments	Acceptance: “Would you accept the COVID19 vaccine when it becomes available?”	63.10%			Good
Gin/2022/USA [22]	January—April 2021	20	Homelessness veterans	Semi-structured interviews	70%	1, Protect own health 2, Protect other 3, Used to get vaccines in military	1, Vaccines were not tested enough 2, Long-term side effects concerns 3, Mistrust in government	Fair
Abramovich/2022/Canada [40]	January—June 2021	91	Youth LGBT + PEH	An adapted version of the Vaccination Attitudes Examination Scale [43]	63.74%	1, Being White	1, Being Black 2, Being racialized versus non-racialized	Good
Meehan/2022/USA [26]	March—June 2021	864	Homeless unshelters	A questionnaire template with over 20 questions to assess COVID-19 vaccine uptake, intention, and associated factors	53.82%	1, Having underlying medical conditions 2, Having previous COVID-19 illness 3, Received health information from hospitals/health centers, religious leaders, and multimedia news sources 4, Protect own health 5, Protect health of family or friends	1, Vaccine was new 2, Received health information from the social media 3, Need more information 4, Side effects concerns 5, Human experiments concerns	Good
Balut/2022/USA [17]	January—April 2021 and July—August 2021		Healthcare and housing service providers’ perspectives of homeless veterans	semi-structured interviews			1, Distrust and cynicism 2, Long vaccine appointment scheduling process 3, Mandating vaccination	Fair
Cox/2022/USA [19]	March 2020—August 2 2021	98	Homeless shelters	Self-report their perceived risk of COVID-19 and intent to receive a COVID-19 vaccine across four different seasonal time point Change intent over time: “Overall, how have your feelings about getting a COVID-19 vaccine changed since beginning of the pandemic (Spring 2020)?”	74.23%	1, Vaccine was safe 2, Vaccine was effective in preventing COVID-19 3, Encouragement from family/friends	1, Mistrust in government and healthcare providers 2, Side effects concerns	Good
Tucker/2022/USA [34]	March—October 2021	125	Young PEH	Acceptance: participants indicated whether they had gotten the COVID-19 vaccine (yes, no) and rated how much they agreed/disagreed with the following two statements: If the COVID-19 vaccine were available to me now (a year from now), I would get the vaccine (1 strongly disagree to 4 strongly agree)	50.4%	1, Being LGBTQ young adults		Good
Finnigan/2022/USA [20]	September 27 -October 8 2021	289	PEH	Acceptance: “Have you received a COVID-19 vaccine?” if the answer was no then there was another question "Do you plan to get vaccinated for COVID-19?”, with answer choices consisting of definitely getting a vaccine, probably getting a vaccine, unsure about getting a vaccine, probably NOT getting a vaccine, and definitely NOT getting a vaccine	74.73%		1, Side effects concerns 2, Did not trust COVID-19 vaccine 3, Did not trust the government	Fair
Currie/2022/Australia [37]	23 September and 28 October 2021	49	PEH	Semi-structured interviews of people who already accepted the vaccine		1, Important to own health 2, Important to community health 3, Someone told to get vaccine 4, Visit loved ones		Fair
Rosen/2022/USA [32]	May—November 2021	4949	PEH	Acceptance: unvaccinated participants were asked if they wanted to get vaccinated and answered yes, no, or not yet Attitudes: list of 13 reasons for vaccine readiness and hesitancy	75.57%	1, $50 gift card 2, Protecting other 3, Outreach staff recommended it	1, Not a top priority 2, Not a afraid of COVID-19 3, Safety concerns 4, Side effects concerns	Fair
Roederer/2022/France [16]	November 15 -December 22 2021	3690	Migrants, homeless shelters, and homeless unshelters	Acceptance: COVID-19 vaccination status was verified via the national vaccine certificate – and interviews for further information	89.26%	1, Protect own health 2, Protect others 3, Vaccine certificate as the motivation (continue to work, travel, etc.)	1, Side effects concerns 2, Fear of injection/serious disease 3, Skepticism about vaccine effectiveness	Good
Grune/2023/Germany [42]	August—April 2022	20	PEH	Semi-structured interviews		1, High risk perception of COVID-19 2, Protect others 3, Continue with normal life 4, Vaccine was effective and safe	1, Side effects concerns 2, Mistrust in government/health systems	Fair
Polla/2022/Italy [38]	June and October 2022	313	PEH	5 section questionnaire: sociodemographic, knowledge about COVID-19, attitudes and beliefs, and COVID-19 vaccination and the reasons for the decision	88.18%	1, Being older 2, Higher knowledge of COVID-19 3, Perceived themselves as a higher risk of the disease	1, Side effects concerns	Good

*Abbreviation*: *PEH* People experiencing homelessness, *VHS* Vaccine Hesitancy Scale developed by World Health Organization

**Table 2:** Characteristics of studies on the acceptance of other kinds of vaccines (not COVID-19)

Author/year/country	Kinds of vaccine	Date of survey	N	Target population	Outcome measures	Acceptance rate	Key findings	Quality
Gennaro/2021/USA [21]	7-vaccine series for children	February 2018 and October 2019	135 children of participants	PEH who have children	Parental vaccine concerns were assessed using items with binary responses		More than one-half (57%) of the participants reported at least 1 concern about childhood vaccines, with 28.1% having 1–2 concerns, 16.3% having 3–4 concerns, and 13.3% having 5 or more concerns	Good
Buechler/2020/USA [18]	Hepatitis A	Early fall and summer of 2018	44	PEH	1. Hesitancy: WorldHealth Organization and the SAGE Working Group on Vaccine Hesitancy [44] 2. Attitudes: Quantitative Vaccine Conspiracy Beliefs Scale [45]	70.45%	Those who hesitated to receive a vaccine cited beliefs in the danger of materials in the vaccines, such as metals or viruses, or the uselessness of vaccines. The only reason given by clients that had outright refused a vaccine was mistrust of the intentions of the provider or manufacturer	Fair
Poulos/2010/Australia [36]	Hepatitis A and B	June 24 2003—February 10 2005	201	Clients attending a medical clinic for homeless	Accept to participate in the vaccination program		Of those with the potential to benefit from vaccination, two clients with unknown immunity and 14 clients known to be non-immune to hepatitis A, B or both, either declined vaccination or did not return	Poor
Partida/2022/USA [29]	Hepatitis A and B	August 1 2018—January 30 2021	86	Homeless shelters	Documentation the proportion of eligible participants who received HAV and HBV vaccination during the vaccine program	Hepatitis A: 53.49% Hepatitis B: 72.09%	Before HCV education, 77.4% of participants felt that it was a “good idea for people living with HCV to be vaccinated against HAV and HBV,” and following education 91.3% agreed with this statement	Fair
Kaplan- Weisman/2018/USA [24]	Herpes Zoster (Shingles)	February 2015—December 2017	84	Homeless shelters ≥ 60 years of age	Accept the vaccine offer during 30-min talk on Zoster vaccine	48.81%	Among 84 participants, 41 accepted the vaccine (include 4 already vaccinated), 39 declined	Good
Story/2014/England [41]	Influenza	July—August 2012	190	Homeless shelters	Could not determine	73.16%	Among 190 eligible people, 73.16% said they would accept vaccine if offered	Good
Nougaire`de/2010/France [35]	Influenza	December 20 2009	250	Homeless shelters	Accept to participate in the vaccination program	46.80%	46.8% of the 250 homeless persons present at the shelter being vaccinated during the one-day campaign	Poor
Metcalfe/2014/USA [28]	Influenza		87	Homeless shelters	1, “Have you ever gotten a flu shot?” 2, “Do you plan to get a flu shot this fall?”	47.13%	47% of the respondents agreed to get a flu shot and 53% weren't planning to take it	Poor

#### Prevalence of COVID-19 vaccine acceptance

**Fig. 2:**
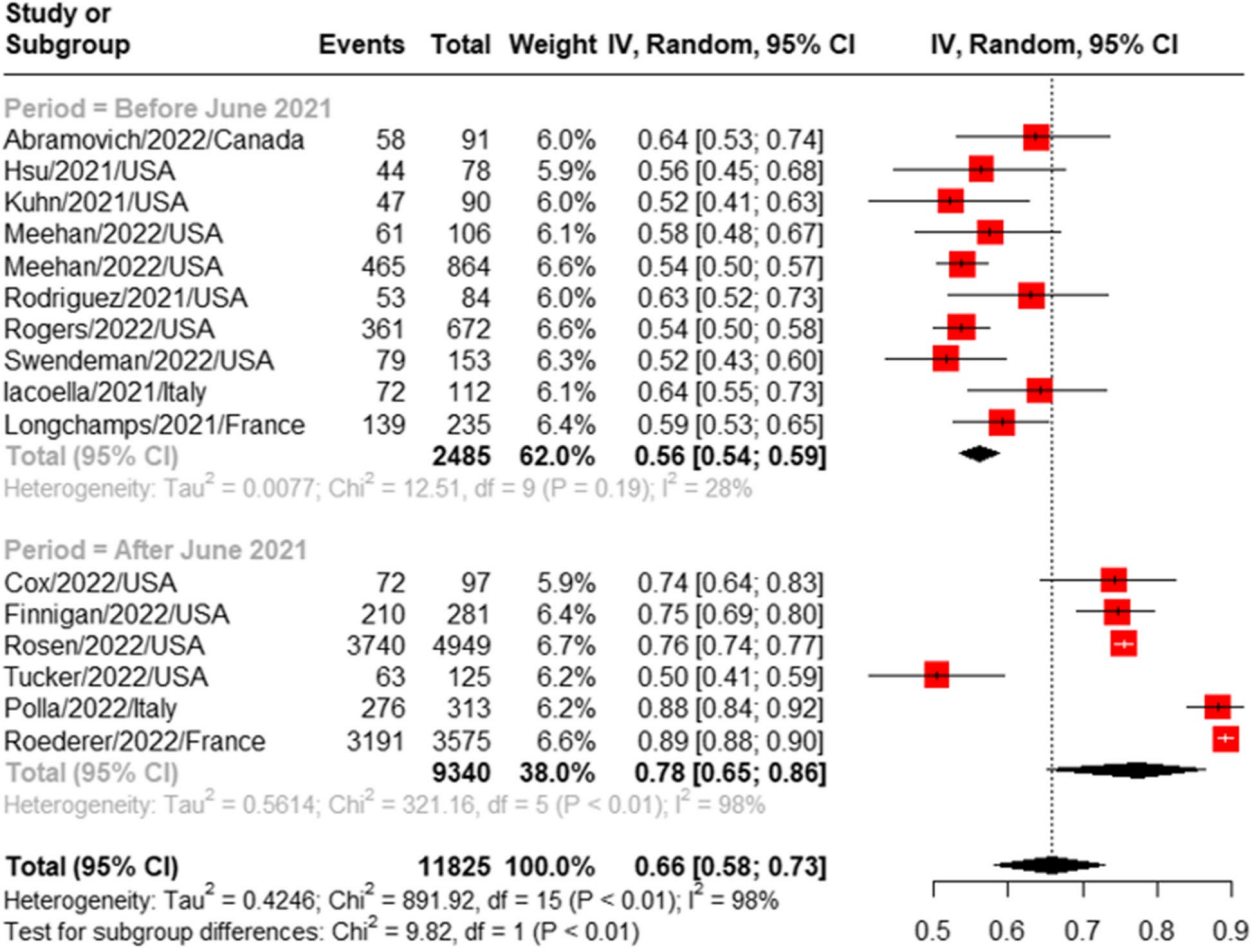
Subgroup meta-analysis of the prevalence of COVID-19 acceptance among people experiencing homelessness

Sixteen studies were pooled for the prevalence of COVID-19 vaccine acceptance [10, 16, 19, 20, 23, 25–27, 30–34, 38–40]. The pooled prevalence of COVID-19 vaccine acceptance was 66% (95%CI: 58%-73%), I^2^ = 98%. A significant increase in the pooled prevalence of vaccine acceptance was noted when comparing the two time periods: before June 2021 (56%, 95%CI: 54%-59%, I^2^ = 28%) [10, 23, 25–27, 30, 31, 33, 39, 40] and after June 2021 (66%, 95%CI: 65%-87%, I^2^ = 98%) [16, 19, 20, 32, 34, 38] (Fig. 2). The heterogeneity was low in the “before June 2021” subgroup. The time interval covariate in the meta-regression model was statistically significantly associated with COVID-19 vaccine acceptance (Table 3).

**Table 3:** Meta-regression results for COVID-19 vaccine acceptance

Covariate	Coefficient β (SE)	z	95% CI	*P* value
Time interval (in day)	0.0024 (0.0007)	3.51	0.0011, 0.0038	0.0004
Intercept	-0.1494 (0.2699)	-0.55	-0.6785, 0.3796	0.5799

#### Prevalence of COVID-19 vaccine uptake

**Fig. 3:**
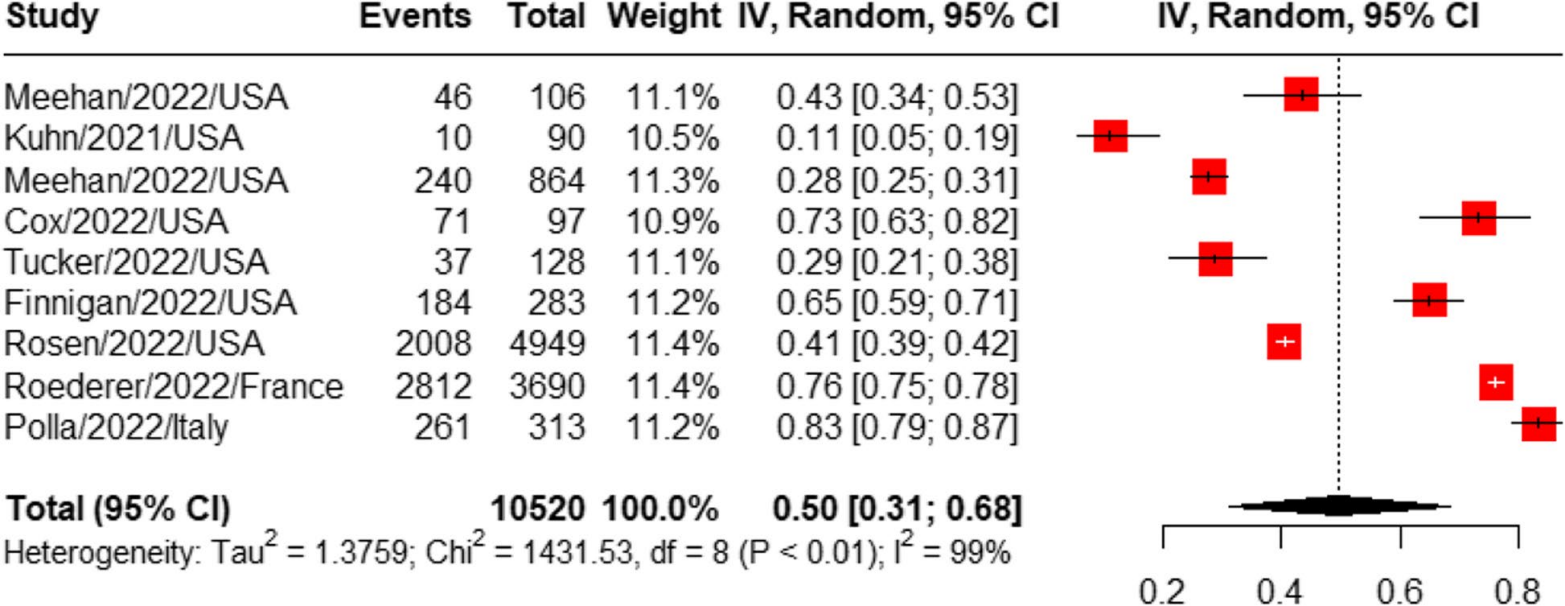
Meta-analysis of the prevalence of COVID-19 vaccine uptake among people experiencing homelessness sorting by the timing of participant recruitment. (The year in the figure was published year)

Nine studies were pooled for the prevalence of COVID-19 vaccine uptake [16, 19, 20, 25–27, 32, 34, 38]. The pooled prevalence of COVID-19 vaccine uptake was 50% (95%CI: 31%-68%), with a high heterogeneity (I^2^ = 99%). See Fig. 3.

#### Factors associated with COVID-19 vaccine acceptance

Analysis of the association of gender and having underlying medical conditions with COVID-19 vaccine acceptance was undertaken in the meta-analysis. Having medical conditions was defined as having chronic diseases or long-term illness.

#### Gender

**Fig. 4:**
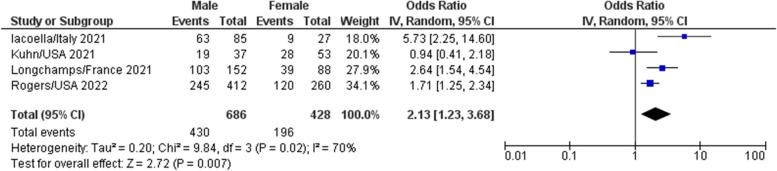
Meta-analysis of the association of gender with COVID-19 vaccine acceptance

Four studies were pooled to determine the association between gender and vaccine acceptance [10, 25, 31, 39]. Males had significantly higher acceptance rates of the COVID-19 vaccine (pooled OR: 2.13, 95%CI: 1.23–2.68, overall effect: Z = 2.72, *P* = 0.007) with a high heterogeneity (I^2^ = 70%). See Fig. 4.

#### Having medical conditions

**Fig. 5:**
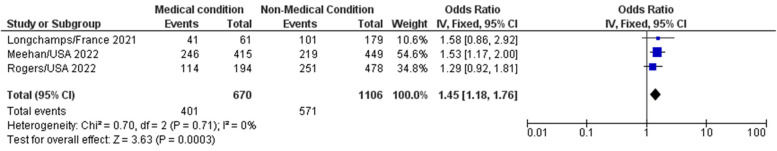
Meta-analysis of the association of medical condition with COVID-19 vaccine acceptance

Three studies were pooled to identify the effect of having medical conditions on vaccine acceptance [10, 26, 31]. Participants with medical conditions showed significantly higher acceptance of COVID-19 vaccination (pooled OR: 1.45, 95%CI: 1.18–1.76, overall effect: Z = 3.63, *P* = 0.0003) with a low heterogeneity (I^2^ = 0%). See Fig. 5.

#### Race

Black/African Canadians and African Americans were less likely to be vaccine acceptance participants (OR = 4.61, 95%CI: 1.42–15) [40] and (aOR = 2.47, 95%CI: 1.57–3.88) [31] respectively.

#### Age

Older age was associated with higher acceptance of the vaccine (OR = 1.04, 95%CI: 1.01–1.07) [38]. A study found that individuals aged between 35 and 65 had higher odds for vaccine acceptance than adults below the age of 35 (aOR = 1.3, 95%CI: 1.0–1.5). The odds were even higher for individuals aged more than 65 than for adults below the age of 35 (aOR = 2.1, 95%CI: 1.3–3.7) [16].

#### Other associated factors

In France, having a higher health literacy level and legal residence were associated with more vaccine acceptance (aOR = 0.38, 95%CI: 0.21–0.68 and aOR = 0.51, 95%CI: 0.27–0.92, respectively) [10]. While those living with others were associated with less vaccine acceptance (aOR = 2.48, 95%CI: 1.17–5.41) [10].

Individuals who had trust in official sources were found to have a significantly higher likelihood of vaccine acceptance (aOR = 0.37, 95%CI: 0.12–1.11) [25]. On the other hand, those who relied more on personal contact were found to have a lower likelihood of vaccine acceptance (aOR = 2.70, 95%CI: 0.93–7.81) [25]. In comparison with an accepting group, a higher percentage of individuals who were hesitant or undecided about getting the vaccine received information about the vaccine through social media (80.0% vs 58.3%; *p* value < 0.001) [46].

Furthermore, individuals who had a high perception of the threat posed by COVID-19 were significantly more likely to be accepted about getting the vaccine (aOR = 0.25, 95%CI: 0.08–0.80) [25]. Conversely, those who engaged in highly protective behavior were less likely to be accepted about getting the vaccine (aOR = 3.63, 95%CI: 1.26–10.47) [25].

#### Attitudes toward the COVID-19 vaccine

**Fig. 6:**
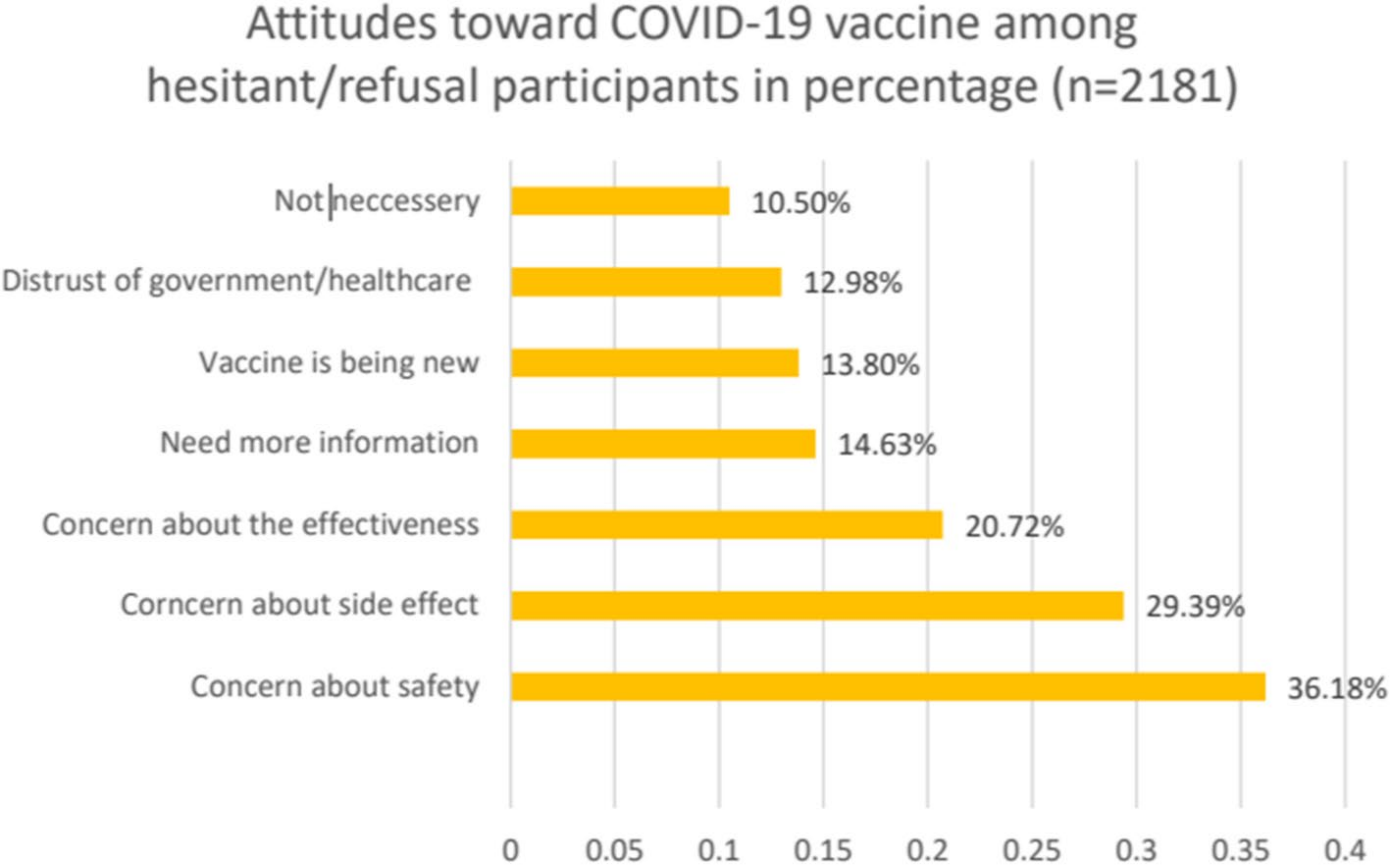
Attitudes toward the COVID-19 vaccine among hesitant and refusal participants (percentage does not add to 100% because people can have multiple attitudes)

We pooled the studies that reported similar attitudes of hesitant/refusal participants (*n* = 2181) toward the COVID-19 vaccine [16, 20, 25–27, 31, 32, 38, 40]. A total of 36.18% reported concerns about the safety of the vaccine [16, 20, 26, 31, 32, 38], 29.39% reported concerns about the side effects of the vaccine [16, 20, 25–27, 32, 38], 20.72% reported concerns about the effectiveness of the vaccine [16, 20, 25, 26, 38], 14.63% said they needed more information [25–27, 31, 32], 13.80% reported concerns about the newness of the vaccine [26, 27, 32], 12.98% did not trust the government and/or the healthcare system [16, 20, 26, 27, 32], and 10.50% felt that the vaccine was unnecessary [16, 32] (Fig. 6).

**Fig. 7:**
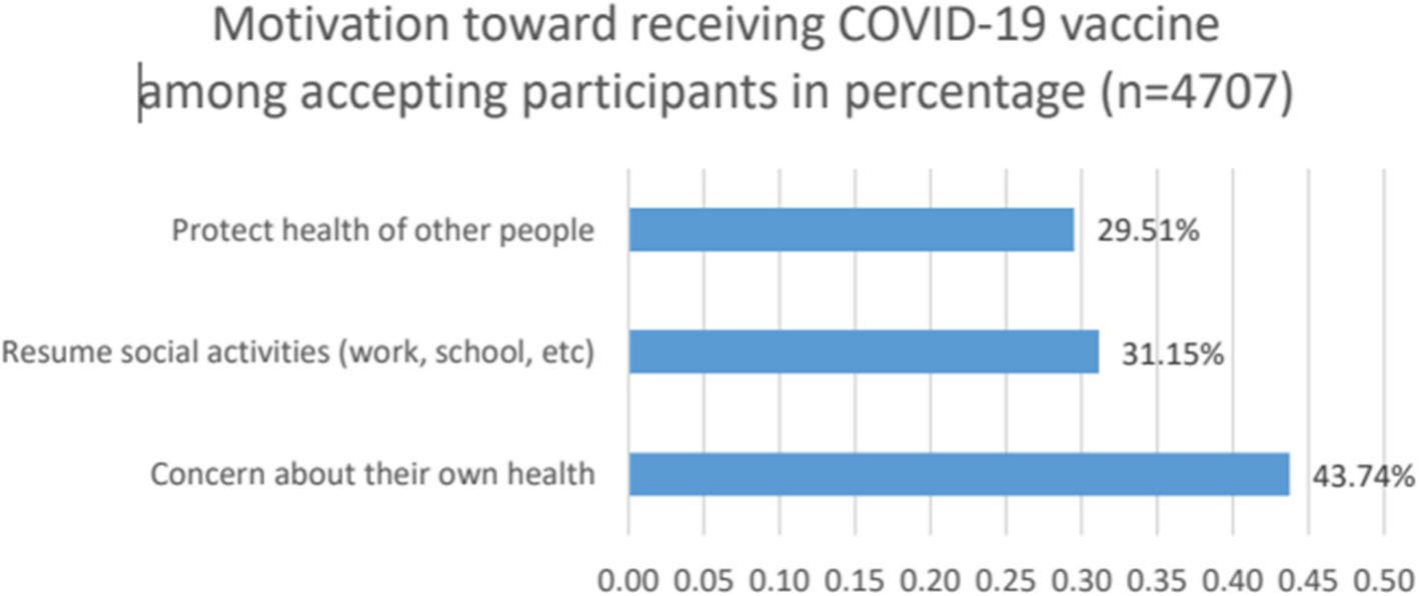
Motivation toward the COVID-19 vaccine among accepting participants (percentage does not add to 100% because people can have multiple attitudes)

Again, we pooled the studies that reported the motivation of participants toward the COVID-19 vaccine (*n* = 4707) [16, 26, 27, 32, 37, 38]. A total of 43.74% reported a willingness to protect themselves [16, 26, 27, 37, 38], 31.15% were motivated by the desire to resume social activities [16, 26, 27, 32, 37], and 29.51% reported a willingness to protect others [16, 26, 27, 32, 37] (Fig. 7). Please refer to Supplementary file 4 for the data on the bar charts.

#### Change in attitudes toward the COVID-19 vaccine over time

Only one study reported how vaccine intentions change over time among PEH [19]. It reported a change in participants' attitudes from 45.3% (*n* = 53) intent to be vaccinated in Spring 2020 to 74.4% (*n* = 87) by August 2021 [19].

#### Prevalence of acceptance of other vaccines (not COVID-19)

**Fig. 8:**
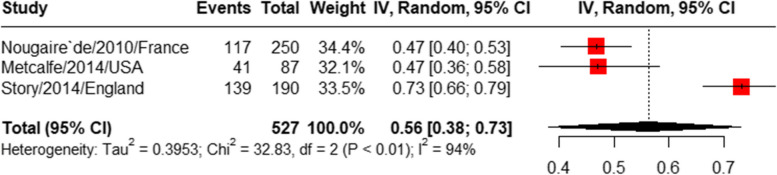
Prevalence of influenza vaccine acceptance among people experiencing homelessness

The pooled prevalence of influenza vaccine acceptance [28, 35, 41] was 56% (95%CI: 38%-73%), with a high heterogeneity (I^2^ = 94%) (Fig. 8). The prevalence of Herpes Zoster vaccine acceptance reported by a single study was 53.57% [24], the prevalence of hepatitis B vaccine acceptance also reported by a single study was 72.09% [29], and hepatitis A reported by two studies was 53.49% [29] and 70.45% [18]. For a summary of key findings from other kinds of vaccines (not COVID-19), please refer to Table 2.

#### Barriers to accessibility to vaccines

Many participants reported being faced with various barriers preventing them from getting vaccinated. Barriers reported by studies include the distance to the vaccination sites [26], lack of transportation [21, 26, 28], lack of information about sites of vaccination [26, 28], inconvenient operating hours of vaccination sites [21, 26], and inability to take off from work to get vaccinated [17, 26].

## Discussion

Since the emergence of COVID-19 in December 2019, healthcare systems have suffered exhaustion, and resources have been depleted despite the strict policies applied by governments to control the pandemic. Eventually, vaccination was an answer to control the pandemic spread. However, vaccine hesitancy varies among different populations depending on ethnicity, socioeconomic status, attitude toward vaccination, and religious beliefs [47]. In this study, we aimed to examine the prevalence of vaccine acceptance and the related factors, especially among PEH, by conducting a systematic review and meta-analysis of 29 studies.

Our meta-analysis revealed that COVID-19 vaccine acceptance rates were significantly higher after June 2021 than in previous periods. Some common attitudes toward the COVID-19 vaccine among hesitant/refusal participants have been reported, including safety concerns, side effect concerns, effectiveness concerns, distrust of governments/healthcare systems, vaccines being new, and vaccines not being necessary. Some common motivations to receive the COVID-19 vaccine are concern about one’s own health, resuming social activities, and protecting other people. Significant factors negatively associated with COVID-19 vaccine acceptance included being female, having no medical condition, being Black individuals residing in Canada and the USA, being younger, and having lower health literacy.

In addition to the COVID-19 vaccine, other kinds of vaccines have also been investigated, including influenza vaccines, hepatitis A & B vaccines, herpes zoster vaccines, and the combined 7-vaccine for children. In this discussion, we will only discuss the COVID-19 vaccine because of the limited information on other kinds of vaccines.

### COVID-19 vaccine uptake compared with the general population

Vaccine uptake rates often reveal the true results of the effectiveness of vaccination strategies. Several studies have been conducted to examine vaccine uptake and coverage among PEH. In comparison to the population in Dane County, USA, PEH had low rates of completing a primary COVID-19 vaccination series (32.0%, 95%CI: 30.3%–33.8%) and receiving a booster when they were eligible (30.8%, 95%CI: 27.8%–33.9%) [48]. The population of Dane County had higher rates of primary vaccination series completion (82.4%, 95%CI: 82.3%–82.5%) and booster vaccination (67.2%, 95%CI: 67.1%–67.4%) [48]. The COVID-19 vaccination completion rates were much lower among PEH in Minnesota, USA, compared to the general population [49]. By September 30, 2021, in Ontario, Canada, (61.4%, 95%CI: 60.8–62.0) of PEH had received their first dose of the COVID-19 vaccine, with (47.7%, 95%CI: 47.0–48.3) having received two doses, while during the same period, 86.6% of Ontario's total adult population had received at least one dose and 81.6% had received two doses [50]. Consistent studies have found that PEH has a lower vaccination rate than the general population. One possible explanation is that PEH may have more challenges in accessing healthcare services, including vaccination clinics. Additionally, vaccine hesitancy or concerns among PEH may also contribute to the lower vaccination rate.

However, there are still some exceptions. In December 2021, Roederer et al. [16] discovered that out of the 3690 PEH and migrants in France, 76.2% (95% CI 74.3–78.1) had received at least one dose of the COVID-19 vaccine. We believe it was not significantly lower than the total number of people vaccinated against COVID-19 in France, with over 80% according to Our World Data [51] in 2023, which covered the entire population. COVID-19 vaccine coverage was found to be high among PEH in Toronto, Canada, with at least one dose being received by 80.4% of the population and 63.6% receiving two or more doses, indicating that advocacy and outreach efforts may have been successful [52]. A study in Italy revealed (83%, 95%CI: 79%-87%) PEH have received the COVID-19 vaccine between June and October 2022 [38]. However, we could not find any papers that reveal high vaccine uptake in the USA. A possible explanation could be that other nations were doing better than the USA for vaccination among PEH.

### COVID-19 vaccine acceptance rate compared with the general population & within the PEH population

#### Before June 2021

According to a systematic review, vaccine acceptance rates in the USA were 56.9% in April, increased to a range of 67.0–75.0% in May, and reached 75.4% in June 2020 [53]. It was higher than our pooled results for vaccine acceptance rate among PEH before June 2021, with the majority of studies conducted in the USA. In Italy, the vaccine acceptance rate was 77.3% in April and 70.8% in June 2020 [53]. The result was also higher than that of a study of PEH in Italy conducted before June 2021. With the available evidence, we can hypothesize that COVID-19 vaccine acceptance among PEH was lower than that among other populations before June 2021.

#### After June 2021

In June 2021, the COVID-19 vaccine acceptance rate was found to be 66.6% in the general population in the USA by a large survey [54]. It was unexpectedly lower than our pool results for the acceptance rate among PEH after June 2021. In our meta-analysis, one study in France and one study in Italy conducted after June 2021 found high acceptance rates with 95% confidence intervals between 84 and 92%, which can be considered very high acceptance rates. Therefore, for the period after June 2021, the COVID-19 vaccine acceptance rate among PEH can be considered similar to that among other populations. But compared to other countries, vaccine uptake and acceptance rates among PEH in the USA are still lower than those in other nations, highlighting the significance of addressing this issue in this country. The variation in different countries can be attributed to many potential factors such as trust in healthcare system, healthcare accessibility, government incentives, outreach strategies, etc.

To better understand the high heterogeneity (I2 = 98%) observed in COVID-19 vaccine acceptance rates after June 2021, four studies were conducted in the USA for this subgroup [19, 20, 32, 34]. Out of these studies, three consistently reported acceptance rates ranging from 74 to 76%. However, one study conducted by Tucker et al. deviated from this trend with a lower acceptance rate of 50% [34]. This discrepancy can be attributed, in part, to the fact that the Tucker et al. study exclusively recruited participants aged 18 to 25. Moreover, two other studies [16, 38] provided evidence that older age was significantly associated with higher vaccine acceptance. Consequently, it led us to formulate a hypothesis with some degree of certainty that younger individuals were less likely to accept the COVID-19 vaccine.

### Hypotheses for high COVID-19 vaccine acceptance rates after June 2021

Acceptance of the COVID-19 vaccine was higher after June 2021. Our study presents a foundation for future studies investigating the trend of the vaccine acceptance rate. A survey in 23 countries found a consistent global trend of higher vaccine acceptance rates in June 2021 than in June 2020 [54]. The announcement of vaccine certification had a strong impact on rising acceptance. Many participants answered that they received vaccination to obtain vaccine certification for their job or to travel abroad; vaccination odds were lower among participants who did not need certification or did not have medical coverage [16]. A study conducted in eight European countries (Austria, Greece, Italy, Norway, Poland, Russia, Spain, and the United Kingdom) revealed that the introduction of COVID-19 certificates led to a significant increase in daily administered vaccine doses in all the countries included along with an immediate positive impact of incentives on vaccine uptake [55]. COVID-19 vaccine uptake is a requirement to continue many social activities, so it could explain the higher acceptance rate over time. However, this also raises some important questions, such as (1) Do people truly believe in vaccines or health care systems? (2) How can trust in healthcare systems be promoted? (3) Ethical considerations surrounding mandatory vaccination.

### Implications for policy and practice

There were some significant factors negatively associated with vaccine acceptance in PEH, such as being female, being Black individuals residing in Canada and the USA, having no medical condition, being younger, and having lower health literacy. Thus, targeted messaging should be implemented in these specific subgroups to address concerns and attitudes toward vaccines. We can establish more education programs to promote the benefits of vaccines among PEH who do not have a medical condition. One approach to promoting health literacy in the homeless population is to provide clear and concise health information in an easily accessible format such as flyers, brochures, or videos. Ensure that outreach and education efforts are culturally competent and respectful of the diverse backgrounds of PEH. This personalized approach can contribute to building trust and creating a welcoming atmosphere of inclusivity.

Some major attitudes among hesitant/refusal participants are concern about vaccine safety, concern about vaccine side effects, the vaccine is not necessary, and do not believe the vaccine will work. Education or outreach education programs should focus on addressing these main concerns. It is important to prioritize efforts that not only emphasize the scientific data supporting vaccine efficacy but also community health.

For the literature review, we found some strategies that have been recommended to improve vaccination rates in PEH. There could be different options for vaccine delivery locations, where staff offer vaccinations in areas convenient to the people. Centers for Disease Control (CDC) recommended vaccination at sites such as shelters, meal services, and encampments for COVID-19 vaccination [56]. Moreover, many studies have suggested using accelerated vaccination schedules for multidose vaccines, which means vaccinating a person at their first appointment [57]. Another strategy could be training staff to work effectively with homeless people if they have not done this before [23]. Education to improve nurses’ attitudes toward PEH may improve the willingness of PEH to present for vaccination [39]. The CDC highlights the need for clear, consistent messaging with PEH about COVID-19 vaccination [56].

A guide by Tucson Pima Collaboration to End Homelessness discussed some core values in discussing COVID-19 vaccine hesitancy among PEH including the right to clear health information, acknowledging vaccine hesitancy as a common concern without judgment, recognizing the potential influence of trauma on healthcare decisions, empowering informed decision-making, and promoting harm reduction for those who choose not to vaccinate [58]. The report emphasized that vaccines should not be mandatory for accessing services or housing assistance.

### Limitations of the study

One of the limitations of the studies addressed in the review is the presence of nonresponse bias. Among the 29 included studies, 23 studies were suspected to have nonresponse bias. Most papers did not disclose how many participants were approached during the study. People who did not respond to the survey might have lower trust in vaccines, have been badly impacted by the pandemic, or have less confidence in the government – so nonresponse bias can be a problem in this situation.

Another limitation is the inconsistency of the outcome measures. The included studies did not use a standardized data form on vaccine acceptance, making it difficult to compare and combine data. For instance, the "not necessary" variable for attitudes was reported in only 2 studies. Therefore, it can be underestimated in the combined attitudes bar chart (Figs. 6 & 7). Additionally, the rates of acceptance reported by the included studies are heterogeneous in many studies which may partly be explained by the characteristics of the settings in which they were conducted, hence the result of this study should be cautiously generalized.

Most papers did not distinguish between data on homeless shelters and homeless non-shelters. In a 2022 report in the USA, only 60% of PEH are residing in shelters or other forms of structured housing [59]. Homeless non-shelters people are considered more vulnerable and could have some significant differences in characteristics compared to homeless shelters people. It was difficult to generalize findings across all homeless populations due to the variability between them in terms of demographics, health status, and social circumstances.

One notable limitation is the limited number of articles considered for meta-analysis of different factors associated with COVID-19 vaccine acceptance. Specifically, when analyzing factors related to vaccine acceptance “gender”, we could only incorporate data from four articles, while for the association of medical conditions with vaccine acceptance, we relied on data from only three articles.

Moreover, we did not include papers that only reported vaccine uptake, so some papers could be missed from this systematic review. Vaccine uptake information is valuable to examine the true vaccine coverage in the population. Thus, our pooled results for vaccine uptake among PEH could be suspected of selection bias.

### Recommendations for future research

We should implement strategies to minimize nonresponse bias to maximize response rates. The best-known strategies to increase response rates are incentives and modes of contact. People are more willing to respond to a survey question when agreement is in the form of payment of a perceived gift or using personal contact could increase response rates [60]. Moreover, social desirability bias should be minimized, and we must ensure that questions are neutral, not inclined toward any particular viewpoint, and do not pose a threat to obtaining unbiased responses.

Future researchers should use a validated and reliable measurement tool to ensure the accuracy and comparability of the outcomes across different populations. We recommended using the outcome measure tools from Roederer et al. [16]. A group of specialists in social determinants of health in homeless and migrant populations were approached to discuss and choose appropriate questions based on the framework designed by the WHO [61]. Except for semi-structured interviews or interviews, we should use a consistent outcome measure tool for surveying.

Researchers should clearly distinguish between data from homeless shelters and homeless non-shelters. This is crucial for understanding the characteristics of these subgroups’ populations. Surveys can include questions that ask respondents if they are currently residing in a shelter or if they are in non-sheltered locations. Researchers can also access administrative records from social service organizations that collect data on PEH.

Finally, we recommended a research question for future studies to address knowledge gaps in this field: How do the attitudes and beliefs of healthcare providers and social service agencies impact vaccine acceptance among PEH?

## Conclusion

Our study found a positive shift in COVID-19 vaccine acceptance rates in PEH after June 2021. Some common concerns among hesitant participants included vaccine safety, side effects, and distrust of authorities. Factors negatively associated with COVID-19 vaccine acceptance included being female gender, having no medical conditions, being Black individuals residing in Canada and the USA, younger age, and lower health literacy. PEH might had lower vaccine uptake rates compared to the general population, attributed to access challenges and hesitancy. Policy recommendations include tailored messaging and accessible vaccination strategies. Further research should minimize bias and distinguish between sheltered and non-sheltered individuals to generalize better to the population.

### Supplementary Information

**Additional file 1: Table S1.**

**Additional file 2: Table S2.**

**Additional file 3: Table S3.**

**Additional file 4: Table S4.**

## Data Availability

The datasets used and/or analysed during the current study are available from the corresponding author on reasonable request.

## Abbreviations

CDC: Centers for Disease Control and Prevention
COVID-19: Coronavirus Disease 2019
PEH: People experiencing homelessness
VHS: Vaccine Hesitancy Scale
WHO: World Health Organization
