# Supplementary material for: Vaccine acceptance, determinants, and attitudes toward vaccine among people experiencing homelessness: a systematic review and meta-analysis
Source: BMC Infect Dis. 2023 Dec 15;23:880. doi: 10.1186/s12879-023-08878-6 (PMC10724884; doi:10.1186/s12879-023-08878-6)
Supplement: Supplementary file 2 — Additional file 2: Table S2. [file 12879_2023_8878_MOESM2_ESM.docx]

**Supplementary Table 2.** Summary of the literature search history.

| **Databases searched** | **Search terms** | **Results on**  **14/01/2023** |
| --- | --- | --- |
| 1. **PubMed** | (homeless OR homelessness OR “ill-housed” OR unhoused OR shelter OR shelters OR unsheltered OR "street people" OR “street person*” OR “insecure housing” OR vagabonds OR hoboes) AND (willingness OR readiness OR hesitancy OR hesitancies OR delay OR delays OR hesitant OR refusal OR refusals OR acceptance OR accept OR reluctance OR agreement OR undecided OR indecisive OR indecisiveness OR indecision OR uncertain OR uncertains OR skeptic OR skeptics OR doubt OR doubts OR decline OR declines) AND (vaccine OR vaccines OR vaccination OR immunization OR immunisation OR immunized OR shot OR shots OR booster) | 186 |
| 1. **Scopus** | (homeless OR homelessness OR “ill-housed” OR unhoused OR shelter OR shelters OR unsheltered OR "street people" OR “street person*” OR “insecure housing” OR vagabonds OR hoboes) AND (willingness OR readiness OR hesitancy OR hesitancies OR delay OR delays OR hesitant OR refusal OR refusals OR acceptance OR accept OR reluctance OR agreement OR undecided OR indecisive OR indecisiveness OR indecision OR uncertain OR uncertains OR skeptic OR skeptics OR doubt OR doubts OR decline OR declines) AND (vaccine OR vaccines OR vaccination OR immunization OR immunisation OR immunized OR shot OR shots OR booster) | 103 |
| 1. **EMBASE** | ((homeless or homelessness or "ill-housed" or unhoused or shelter or shelters or unsheltered or "street people" or "street person*" or "insecure housing" or vagabonds or hoboes) and (willingness or readiness or hesitancy or hesitancies or delay or delays or hesitant or refusal or refusals or acceptance or accept or reluctance or agreement or undecided or indecisive or indecisiveness or indecision or uncertain or uncertains or skeptic or skeptics or doubt or doubts or decline or declines) and (vaccine or vaccines or vaccination or immunization or immunisation or immunized or shot or shots or booster)) | 94 |
| 1. **Web of Science** | (homeless or homelessness or "ill-housed" or unhoused or shelter or shelters or unsheltered or "street people" or "street person*" or "insecure housing" or vagabonds or hoboes) and (willingness or readiness or hesitancy or hesitancies or delay or delays or hesitant or refusal or refusals or acceptance or accept or reluctance or agreement or undecided or indecisive or indecisiveness or indecision or uncertain or uncertains or skeptic or skeptics or doubt or doubts or decline or declines) and (vaccine or vaccines or vaccination or immunization or immunisation or immunized or shot or shots or booster) | 159 |
| 1. **Google Scholar** | allintitle: (homeless OR homelessness OR “ill-housed” OR unhoused OR shelter OR shelters OR unsheltered OR "street people") (hesitancy OR hesitancies OR refusal OR delay OR hesitant OR acceptance OR accept OR reluctance) (vaccine OR vaccines) | 13 |
| 1. **Cochrane** | (homeless OR homelessness OR “ill-housed” OR unhoused OR shelter OR shelters OR unsheltered OR "street people" OR “street person*” OR “insecure housing” OR vagabonds OR hoboes) AND (willingness OR readiness OR hesitancy OR hesitancies OR delay OR delays OR hesitant OR refusal OR refusals OR acceptance OR accept OR reluctance OR agreement OR undecided OR indecisive OR indecisiveness OR indecision OR uncertain OR uncertains OR skeptic OR skeptics OR doubt OR doubts OR decline OR declines) AND (vaccine OR vaccines OR vaccination OR immunization OR immunisation OR immunized OR shot OR shots OR booster) in Title Abstract Keyword | 12 |
| 1. **ClinicalTrials.gov** | "Vaccine Hesitancy" OR "Vaccine Refusal" AND (homeless OR homelessness) | 25 |
| 1. **metaRegister of Controlled Trials (mRCT)** | (homeless OR homelessness OR "ill-housed" OR unhoused OR shelter OR shelters OR unsheltered OR "street people" OR "street person*" OR "insecure housing" OR vagabonds OR hoboes) AND (willingness OR readiness OR hesitancy OR hesitancies OR delay OR delays OR hesitant OR refusal OR refusals OR acceptance OR accept OR reluctance OR agreement OR undecided OR indecisive OR indecisiveness OR indecision OR uncertain OR uncertains OR skeptic OR skeptics OR doubt OR doubts OR decline OR declines) AND (vaccine OR vaccines OR vaccination OR immunization OR immunisation OR immunized OR shot OR shots OR booster) | 0 |
| **Total** |  | 592 |

Duplicate: 240

Screening: 352
